# Silver Nanoparticles Alone or in Combination with Calcium Hydroxide Modulate the Viability, Attachment, Migration, and Osteogenic Differentiation of Human Mesenchymal Stem Cells

**DOI:** 10.3390/ijms24010702

**Published:** 2022-12-31

**Authors:** Almaha S. Algazlan, Nihal Almuraikhi, Manikandan Muthurangan, Hanan Balto, Fahd Alsalleeh

**Affiliations:** 1 Restorative Dental Sciences, College of Dentistry, King Saud University, Riyadh 11461, Saudi Arabia; 2 Stem Cell Unit, Department of Anatomy, College of Medicine, King Saud University, Riyadh 11461, Saudi Arabia

**Keywords:** silver nanoparticles, characterization, endodontic regeneration, toxicity, triple antibiotic paste, calcium hydroxide, stem cells, osteogenic differentiation

## Abstract

This study aimed to evaluate the effect of silver nanoparticles (AgNPs) alone or in combination with calcium hydroxide (Ca(OH)_2_) on the proliferation, viability, attachment, migration, and osteogenic differentiation of human mesenchymal stem cells (hMSCs). Different concentrations of AgNPs alone or mixed with Ca(OH)_2_ were prepared. Cell proliferation was measured using AlamarBlue, and hMSCs attachment to dentin disks was evaluated using scanning electron microscopy. Live–dead imaging was performed to assess apoptosis. Wound healing ability was determined using the scratch-migration assay. To evaluate osteogenic differentiation, the expression of Runt-related transcription factor (RUNX2), Transforming growth factor beta-1 (TGF-β1), Alkaline Phosphatase (ALP), and Osteocalcin (OCN) were measured using real-time reverse transcriptase polymerase chain reaction. ALP staining and activity were also performed as indicators of osteogenic differentiation. AgNPs alone seemed to favor cell attachment. Lower concentrations of AgNPs enhanced cell proliferation. AgNP groups showed markedly less apoptosis. None of the medicaments had adverse effects on wound closure. The expression of TGF-β1 was significantly upregulated in all groups, and OCN was highly expressed in the AgNP groups. AgNPs 0.06% showed the most enhanced ALP gene expression levels, activity, and marked cytochemical staining. In conclusion, AgNPs positively affect hMSCs, making them a potential biomaterial for various clinical applications.

## 1. Introduction

Treatment of immature necrotic teeth is considered one the most challenging procedures in Endodontics. Long-term calcium hydroxide (Ca(OH)_2_) apexification was the original proposed treatment modality for immature necrotic teeth [1,2,3]. Due to the increase in root fracture and the need for high patient compliance, apexification later evolved into a one-step method with mineral trioxide aggregate (MTA) [4,5,6,7,8]. Even though both methods have comparable success rates, neither can achieve continued root development or restoration of pulp tissues [9,10].

This led to a paradigm shift towards endodontic regeneration. Regenerative endodontic procedures (REPs) are defined as: biologically based procedures designed to physiologically replace damaged tooth structures, including dentin and root structures, as well as cells of the pulp–dentin complex [11,12]. The primary goal of endodontic treatment is elimination of disease, but REPs also aim to gain root thickness and length, and restore the functions of the pulp-dentin complex [13,14]. In addition to the classic tissue engineering triad (stem cells, scaffolds, and growth factors), REPs have been found to rely on a new fourth pillar: root canal disinfection [15,16]. 

The nature of dental pulp (endodontic) pathosis was proven to be microorganism-mediated [17,18]. Therefore, root canal disinfection is a fundamental step in endodontics, especially in REPs. A recent systematic review showed that 79% of failed regeneration cases presented with persistent infection [16]. Given the complex microbiota of the root canal system, many intracanal medicaments have been proposed for endodontic disinfection, such as calcium hydroxide [Ca(OH)_2_] and triple antibiotic paste [TAP]. Despite the clinical efficacy of these medicaments, many limitations have been reported; including toxicity, discoloration, and antibiotic resistance [19,20,21,22,23].

Ca(OH)_2_ is one of the most commonly used endodontic medicaments, and its antibiofilm effect is well-documented [21,24,25,26]. Nevertheless, it has limited action against some key organisms in endodontic pathosis, such as *Fusobacterium nucleatum*, *Enterococcus faecalis*, and *Candida albicans* [21,22,26,27,28,29,30]. Despite being promoted as a “biological” material, multiple reports of the toxicity of Ca(OH)_2_-containing materials have been published [31,32,33,34]. This harmful effect poses a problem, mainly when this material, which is usually confined within the root canal system, intrudes into the periapical tissues during endodontic procedures or perforation repairs [35]. 

Nanomedicine is considered one of the breakthroughs of the 21st century. Nanoparticles, with their improved physical-chemical properties, such as nanoscale-small sizes, large surface area, and high surface reactivity, have opened new horizons of treatment and prevention [36,37]. Silver has a long history of being used to prevent infection [38,39]. In the 1800s, silver was used to avoid wound contamination in burn victims [40] and was used as an oral antibacterial solution before the introduction of antibiotics [41]. Nowadays, in dentistry, silver nanoparticles (AgNPs) are gaining popularity and have been used in many forms, including irrigants [42,43,44,45,46], intracanal medicaments [42,44,47,48], dental adhesives [49,50], and as an additive within sealers/restorative materials [51,52,53,54]. Studies reported that AgNPs could be a promising alternative medicament, with their broad-spectrum antibiofilm action and ability to reach complex anatomies [37,55,56]. Moreover, the antibacterial action of AgNPs was reported to be even stronger when incorporated with Ca(OH)_2_ [44,47,48,57,58]. 

The potential toxicity of AgNPs is a concern. However, this is dependent on many factors, and the mechanism is poorly understood [59,60]. An in vitro and in vivo study reported that smaller AgNPs caused more cytotoxicity, oxidative stress and pulmonary inflammation than larger AgNPs [61]. In contrast, another investigation reported that AgNP size did not play a role in the viability of the tumor cells studied [62]. Moreover, AgNPs in higher concentrations ranging from 0.1–5% suppressed neural outgrowth and reduced cell viability when evaluated on rat cortical cell cultures [63]. The differences in AgNP size, dose, concentration, duration of action, and the cell lines studied might explain the variations in the literature in regard to the cytotoxicity of AgNPs [59]. Nevertheless, it is crucial to identify an adequate concentration of AgNPs, with or without Ca(OH)_2_, which can eradicate microorganisms without hindering the healing process or harming the surrounding stem cells. Therefore, this study aimed to evaluate the effect of AgNPs alone or in combination with Ca(OH)_2_ on the proliferation, viability, attachment, migration, and osteogenic differentiation of hMSCs.

## 2. Results

**Figure 1:**
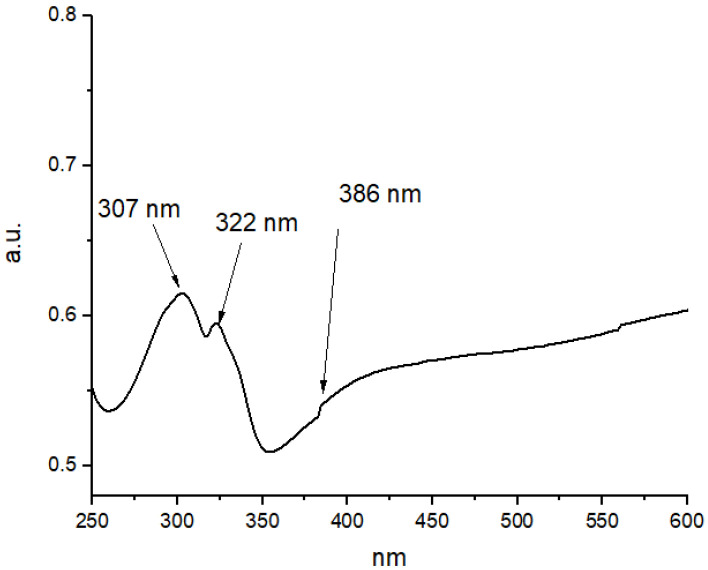
Absorption spectrum of the AgNPs.

For UV-Vis, an absorption band at around 190–1100 nm wavelength was used. The sample presented the characteristic surface plasmon of silver nanoparticles, and showed three peaks: two narrow bands at 307 nm and 322 nm, and a broader band at 386 nm. The interband transitions of electrons caused an absorption that was detected over a broad range (approximately 250 nm–325 nm) (Figure 1). The intensity of the localized surface plasmon resonance (LSPR) band continued to increase, shifted to a shorter wavelength, and narrowed down with further UV irradiation.

**Figure 2:**
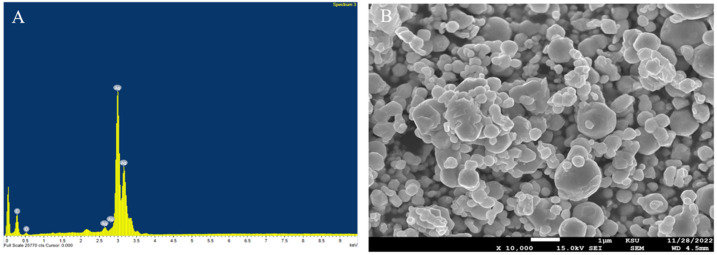
(**A**) EDS compositional analysis of elements, (**B**) SEM image showing the distribution of spherical and semi-identical particles of AgNPs.

Compositional analysis of the elements was conducted using EDS to confirm the existence and purity of AgNPs. A noticeable difference in the percentages of carbon (C), oxygen (O), and silver (Ag) elements is shown in Figure 2A. Generally, around 3 keV, the optical absorption peak of silver appeared. Some particles got oxidized and agglomerated by forming silver oxide, as indicated by the presence of oxygen, but the quantity was very small. SEM imaging showed crystalline particles that were spherical, semi-identical in shape, and well distributed (Figure 2B).

**Figure 3:**
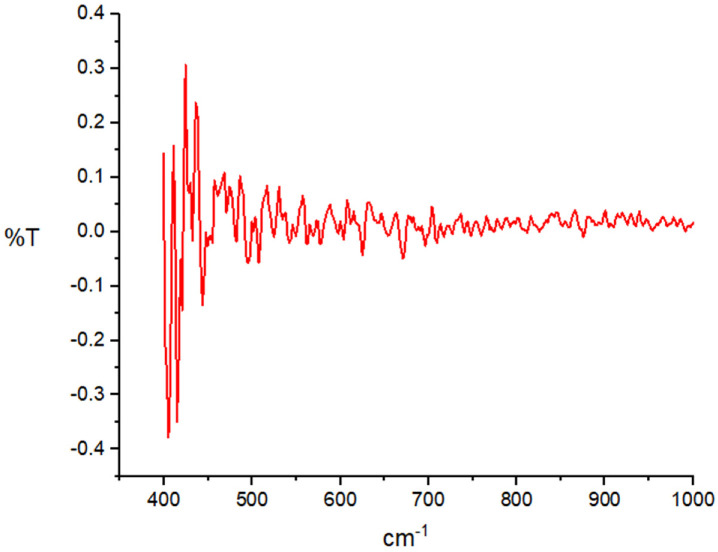
FTIR spectrum analysis of AgNPs.

FTIR helps analyze multi-component systems and provides necessary information pertaining the material’s phase composition and type of interactions between various compounds and polymers. In this study, FTIR was employed to detect the purity of AgNPs. The FTIR spectrum (Figure 3) showed the fingerprint zones at the 500–400 cm^−1^, which are identical to the vibration frequency of Ag–O ionic bond groups. 

### 2.1. AgNPs Favor Cell Attachment

**Figure 4:**
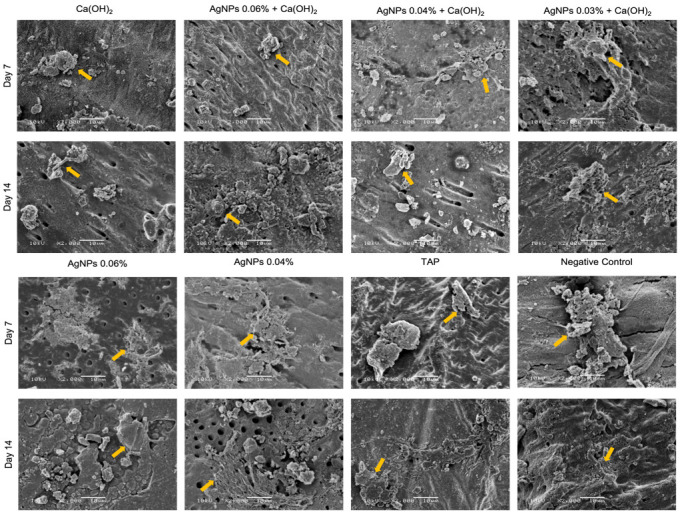
Representative SEM images of human mesenchymal stem cells grown on dentin disks that were immersed in the test medicaments for either 7 or 14 days. Yellow arrows point to mesenchymal stem cells. Scale bar: 10 µm, ×2000 magnification. [Ca(OH)_2_: Calcium hydroxide, AgNPs: Silver nanoparticles, TAP: Triple antibiotic paste].

Representative SEM images are shown in Figure 4. HMSCs were grown on dentin disks immersed in the test medicaments for either 7 or 14 days. Calcium hydroxide and TAP samples showed altered cell morphology (rounder) and more debris, while AgNPs alone favored cell attachment. The combination groups (Ca(OH)_2_ + AgNPs) exhibited elongated cells that appeared adhered to dentin. For all groups, day 14 samples showed a higher number of cells that are more widely distributed than day 7 samples.

**Figure 5:**
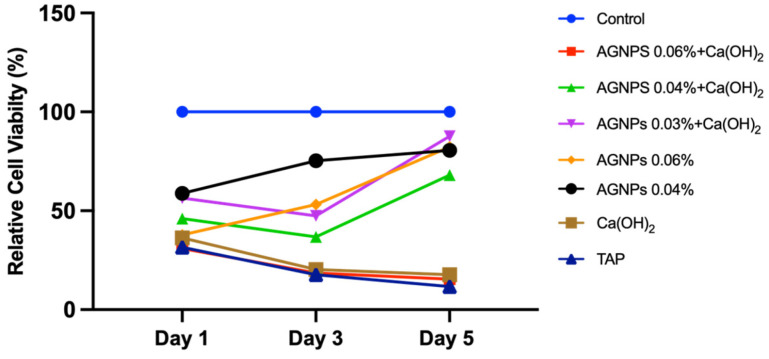
Relative cell proliferation compared with control (cells without treatment) as measured by AlamarBlue assay on days 1, 3, and 5 post-treatment. [Ca(OH)_2_: Calcium hydroxide, AgNPs: Silver nanoparticles, TAP: Triple antibiotic paste].

Lower Concentrations of AgNPs Enhance Cell Proliferation:

AlamarBlue results are shown in Figure 5. All groups exhibited a trend towards increased cell proliferation on day 5, except for [AgNPs 0.06% + Ca(OH)_2_], Ca(OH)_2,_ and TAP. The highest concentration of the combination groups [AgNPs 0.06% + Ca(OH)_2_] showed a gradual reduction in cell proliferation in a similar manner to Ca(OH)_2_ and TAP (*p* < 0.05). The [AgNPs 0.03% + Ca(OH)_2_] group had significantly higher cell proliferation across all time points when compared with treatment controls Ca(OH)_2_ and TAP. On day 5, the proliferation of the [AgNPs 0.03% + Ca(OH)_2_] and [AgNPs 0.06%] groups increased dramatically, showing similar cell proliferation to the control group.

### 2.2. AgNPs Induce Less Apoptosis

**Figure 6:**
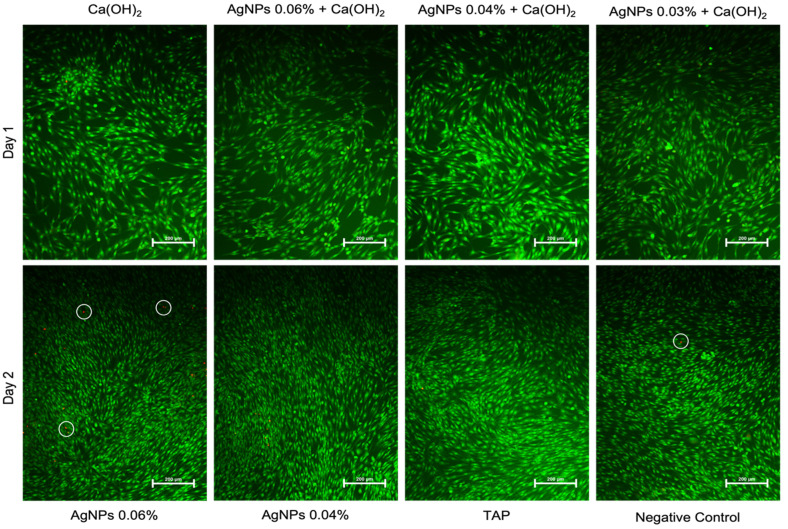

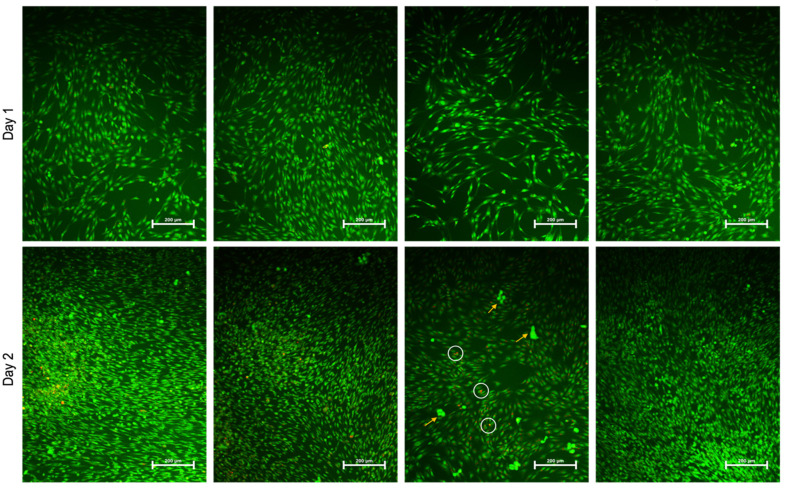
Representative fluorescence images (20×) of hBMSCs treated with the test medicaments, cells were stained with acridine orange/ethidium bromide AO/EtBr and micrographs were taken after 24 and 48 h. White circles on red dots indicate dead (necrotic) cells, yellow arrows point to condensed green structures that indicate chromatin fragments and apoptotic bodies. [Ca(OH)_2_: Calcium hydroxide, AgNPs: Silver nanoparticles, TAP: Triple antibiotic paste].

**Figure 7:**
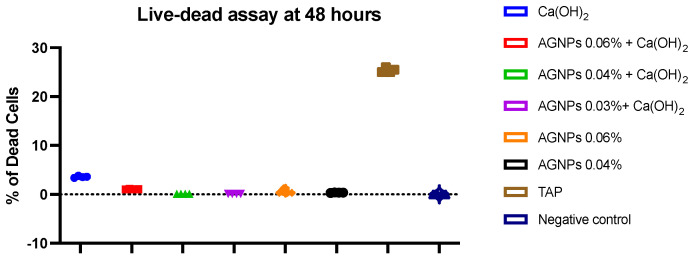
Percentage of dead to live cells on day 2. Data are representative of three replicas for each experimental condition, where more than 4 images were captured per condition.

Representative images from the acridine orange/ethidium bromide (AO/EtBr) staining on days 1 and 2 post-treatment are shown in Figure 6, and the calculated percentages of dead cells on day 2 are shown in Figure 7. On day 2, increased cell proliferation was evident in all groups. In addition, cells treated with TAP exhibited more apoptotic bodies (condensed chromatin) and a higher number of necrotic cells (red dots) than the remaining groups (Figure 6 and Figure 7). However, these changes were mainly observed on Day 2 (Figure 6 and Figure 7). 

**Figure 8:**
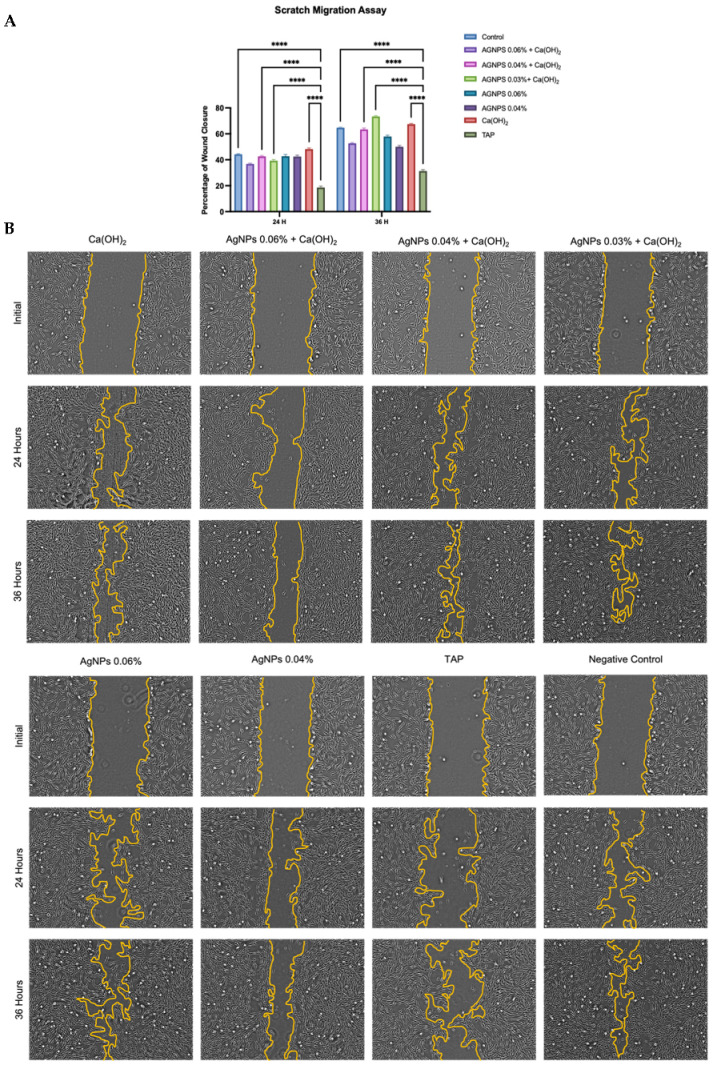
Results of the scratch migration assay at 0, 24, and 36 h. Percentages of wound closure are shown in (**A**), and representative images are shown in (**B**). Asterisks (****) indicate statistical significance (*p* < 0.05). Scale bar: 50 µm [Ca(OH)_2_: Calcium hydroxide, AgNPs: Silver nanoparticles, TAP: Triple antibiotic paste].

AgNPs Have No Adverse Effects on Wound Closure:

Wound closure was quantified, and the results are shown in Figure 8A. Results indicate that the wound healing percentages significantly increased with time for each group, and none of the medicaments decreased the migration ability at any time. On the other hand, TAP exhibited the least migration ability among all groups. This difference was significant compared with the control and 3 experimental groups, as shown in Figure 8A. The combination group AgNPs 0.03%+ Ca(OH)_2_ appeared to have the highest percentage of wound closure among all groups. However, this was only significant when compared with TAP. Representative images are shown in Figure 8B.

**Figure 9:**
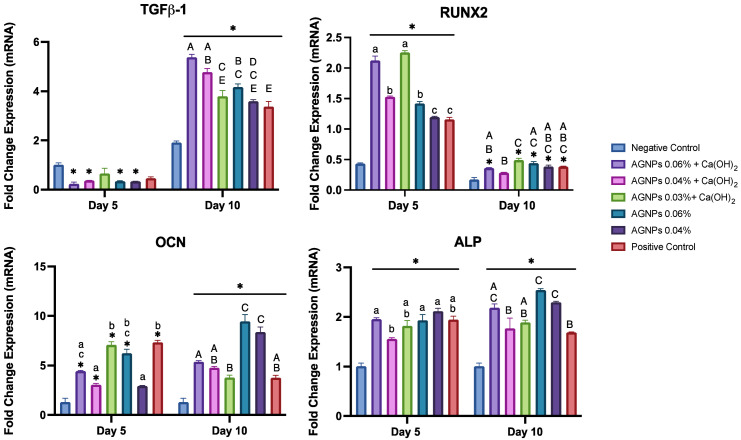
Results of qRT-PCR on days 5 and 10 after osteoblastic induction. Gene expression levels of TGF-β1, ALP, and OCN were analyzed and normalized to GAPDH. Data are presented as mean ± SEM from three separate experiments. Different letters indicate a significant difference (*p* < 0.05). Bars that have the same letter are not significantly different. Small letters are for day 5 and capital letters are for day 10 values. Asterisks (*) indicate statistical significance when compared with negative control (Negative control: cells in basal medium, Positive control: cells in osteogenic medium). [TGF-β1: Transforming growth factor beta, Runx2: Runt-related transcription factor, ALP: Alkaline Phosphatase, OCN: Osteocalcin, Ca(OH)_2_: Calcium hydroxide, AgNPs: Silver nanoparticles].

All Medicaments Enhanced Osteogenic Marker Expression: 

For data simplification, the gene expressions of the test medicaments only (AgNPs in combination and alone) are presented in Figure 9. On day 10 of osteoblastic differentiation, the expression of TGF-β1 was significantly upregulated in all groups compared with their levels on day 5 and the negative control. On day 10, the most potent combination groups [(AgNPs 0.06% and 0.04%+ Ca(OH)_2_)] showed the highest expression of TGF-β1, with a 22 (*p* < 0.05) and 12.7-fold increase, respectively. Conversely, the two lowest concentrations of AgNPs (in combination or alone) resulted in expression levels comparable to the positive control.

The Runx2 expression was significantly upregulated on day 5 compared with the negative control (*p* < 0.05). The mixture of AgNPs + Ca(OH)_2_ showed higher levels compared with the AgNPs alone and the positive control. The AgNPs 0.03% + Ca(OH)_2_ showed the highest expression levels of Runx2 on both day 5 and day 10, compared with the rest of the groups. All groups exhibited a down-regulated expression of Runx2 with time (*p* < 0.05). 

On day 10, the expression of OCN showed the most dramatic increase in the AgNPs 0.06% (1.5-fold) and AgNPs 0.04% (2.84-fold) groups when compared with their levels on day 5 (*p* < 0.05). These two groups also upregulated the day 10 expression of OCN significantly more than all the other groups (*p* < 0.05). The OCN expression levels on day 10 were not very different when comparing the combination groups (Ca(OH)_2_ and AgNPs) to the positive control.

All groups significantly upregulated ALP on days 5 and 10 compared with the negative control. On day 10, the expression of ALP was significantly elevated in three groups [AgNPs 0.06%, AgNPs 0.04% and AgNPs 0.06% + Ca(OH)_2_] (*p* < 0.05). The remaining medicaments exhibited similar expression levels to the positive control.

**Figure 10:**
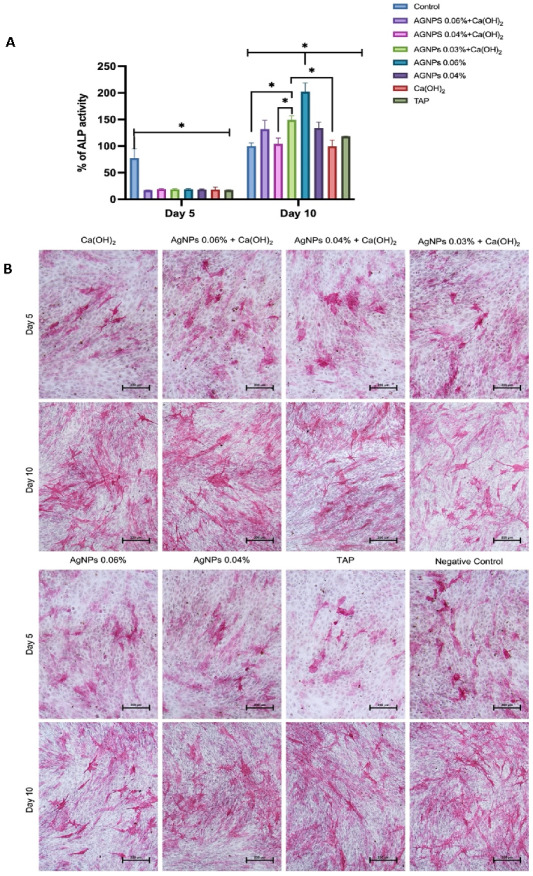
(**A**): Quantification of ALP activity in treated-hBMSCs on days 5 and 10 of osteoblastic differentiation. Control is cells in osteogenic medium. Data are presented as mean percentage of ALP activity ± SEM. Asterisks (*) indicate statistical significance (*p* < 0.05). (**B**): Representative alkaline phosphatase (ALP) staining of hBMSCs treated with the test medicaments, images were taken on days 5 and 10 of osteoblastic differentiation. [Ca(OH)_2_: Calcium hydroxide, AgNPs: Silver nanoparticles, TAP: Triple antibiotic paste].

AgNPs at 0.06% Enhance Alkaline Phosphatase Activity and Staining:

All the tested molecules upregulated the ALP activity with time (Figure 10A), concordant with an increase in the intensity of the ALP cytochemical staining (Figure 10B). Day 5 levels of ALP activity were similar in all groups. However, on day 10, AgNPs 0.06% showed the highest ALP activity, and this increase significantly differed from all remaining groups.

## 3. Discussion

Microorganisms, especially in biofilm form, are the primary culprits of endodontic pathosis [17,18,64,65]. Biofilm microorganisms are highly resistant to disinfecting agents used in endodontic treatment; therefore, combined agents were recommended to enhance their activities and to balance their deficiencies against persistent infections [66,67,68]. Accordingly, the combination of AgNPs with Ca(OH)_2_ has been investigated and showed a synergistic effect compared with the use of Ca(OH)_2_ alone [44,47,48,57]. The ideal intracanal medicament must be biocompatible and ensures the survival of the surrounding stem cells [69]. In this study, we investigated the effect of previously optimized concentrations of AgNPs, alone and in combination with Ca(OH)_2_, on the proliferation, viability, attachment, migration, and differentiation of human bone marrow mesenchymal stem cells [48].

As a model for human bone marrow stem cells (hBMSCs), a telomerized hMSC line (hMSC-TERT) was employed in this study. This cell line was generated by overexpression of the human telomerase reverse transcriptase gene (hTERT). It manifests all the characteristics and genetic markers of primary hBMSCs, including multipotency, high differentiation capability, and unlimited self-renewal [70,71]. Bone marrow stem cells have similar features to dental pulp stem cells, and have exhibited multipotency and strong differentiation abilities, making them a possible source for the regeneration of dental tissues [72,73,74,75].

The toxicity of AgNPs is frequently cited as a concern and depends on many factors, including the AgNP concentration [59]. The cytotoxicity of AgNPs is directly proportional to their concentration, and several concentrations of AgNPs were tested in the literature. 0.01% AgNPs is the lowest concentration that exhibited good antibacterial properties in published studies [42,57,76,77] with no significant changes in tooth color [78]. Furthermore, the concentrations of tested AgNPs range up to 0.15%, which has been used as an additive in endodontic sealers and showed improved antibacterial effects with minimal discoloration [53,79].

When used as an irrigant at concentrations up to 0.025%, AgNPs demonstrated low cytotoxicity and genotoxicity against L929 fibroblasts [80]. Additionally, using L929 fibroblasts, Takamiya et al. evaluated the cytotoxicity of silver nanoparticles synthesized with ammonia and polyvinyl pyrrolidone at different concentrations ranging from 0.00001–0.01% [81]. They also assessed the reaction of subcutaneous connective tissue of Wistar rats to AgNPs and reported that AgNPs were not cytotoxic at 0.0025% or lower. [81]. An in-vivo study concluded that 0.0047% nanosilver dispersions were biocompatible compared with 2.5% NaOCl solution when tested on fibrous connective tissues of rats [55]. In the present study, optimized concentrations were used [48]. The AlamarBlue results presented herein showed that all groups exhibited a trend towards increased cell proliferation on day 5 (Figure 5) and that the lowest concentrations had significantly higher cell proliferation, which agrees with the previously mentioned studies.

Cell attachment and morphology were also examined using SEM. AgNPs alone seemed to favor cell attachment, and the combination groups showed elongated cells adhering to the dentin surface. Cells treated with Ca(OH)_2_ and TAP had altered morphology, consistent with the AlamarBlue results, where these two groups exhibited less viable cells. The detrimental effect of TAP on cell attachment is in agreement with a previous study [82].

The size and shape of the nanoparticles are other essential features that affect their toxicity [59,83,84,85,86]. For example, Liu et al. reported that 5 nm AgNPs were more cytotoxic than 20 nm AgNPs when tested against four cell lines [83]. On the other hand, another study reported that spherical-shaped particles did not induce adverse effects on cells [84]. Therefore, the present study utilized spherical silver nanoparticles with a 20 nm size.

Cell migration is a critical step in pulp wound healing, and TGF-β1 plays a pivotal role in this process. TGF-β1 is released early in the healing stages and acts in the recruitment of inflammatory cells and the expression of essential proteins that form the extracellular matrix [87]. In this study, the gene expression of TGF-β1 on day 10 was significantly upregulated in all groups compared with their levels on day 5 and the negative control (Figure 9). This was also in accordance with the wound-healing assay, where the migration ability was enhanced in cells treated with AgNPs, especially combined with Ca(OH)_2_ (Figure 8). The results agreed with previous reports, which observed that AgNPs and Ca(OH)_2_ increased migration rate and wound healing [85,88,89,90].

In addition to its role in wound healing, TGF-β1 is a crucial osteogenic marker. It regulates osteoblastogenesis and bone formation and increases osteoprogenitor cells through chemotaxis [86,91]. TGF-β1 is also a known inducer of odontoblastogenesis and mineralization of dental tissues [92,93,94]. The upregulation of TGF-β1 gene expression highlights the potential favorable effect of the tested medicaments on the osteogenic differentiation and mineralization.

Runx2 is considered the master-switch of osteoblastic differentiation. It is highly expressed in preosteoblasts, reaches the peak level in immature osteoblasts, and is reduced with osteoblastic maturation [95]. The results in this experiment exhibited the classic behavior of Runx2, where its expression levels were initially upregulated, but later decreased at 10 days. Moreover, groups containing Ca(OH)_2_ showed higher levels of Runx2 expression, which is consistent with previous reports [96,97].

Other assays were used to evaluate the effect of the tested medicaments on osteogenic differentiation. The expression of ALP and OCN was quantified using RT-PCR (Figure 8), and the ALP activity was also quantitatively and qualitatively assessed (Figure 9). ALP is one of the earliest osteoblastic differentiation markers, while OCN is a relatively late marker [98,99]. The q-RT-PCR indicated that AgNPs significantly upregulated the expression of ALP and OCN after 10 days of osteoblastic differentiation and that AgNPs 0.06% had the highest expression levels (Figure 5). Consistently, AgNPs 0.06% showed the most increased ALP activity (Figure 6) and intense cytochemical staining (Figure 7). This is in agreement with previous studies [100,101,102,103]. However, one study reported that AgNPs decreased ALP and OCN expression, which could be attributed to the differences in cell lines and periods [104].

## 4. Materials and Methods

The study protocol was reviewed and approved by the Institutional Review Board (IRB No. E-20-5025) and the College of Dentistry Research Center (CDRC No. PR 0116).

### 4.1. Characterization of AgNPs 

Silver nanoparticles (20 nm) were obtained as a powder from Zhengzhou Dongyao Nano Materials Company. Their size, shape, size, distribution, and aggregation were all examined using ultraviolet–visible (UV-Vis) spectroscopy (PerkinElmer, Norwalk, CT, USA), scanning electron microscopy (JSM-7610F-Field Emission Scanning Electron Microscope-JEOL-Japan), and the Nicolet 6700 Fourier-transform infrared (FTIR) Spectrometer (Thermo Fisher Scientific, Madison, WI, USA). Moreover, the existence and purity of the AgNPs were confirmed by compositional analysis of the elements using energy dispersive spectroscopy (EDS).

### 4.2. Medicament Selection and Preparation

The concentrations of AgNPs were selected based on the minimum inhibitory concentration (MIC) and minimum bactericidal concentration (MBC) of a previous study [48], in which the chosen medicaments exhibited desirable antibacterial effects. 

The selected medicaments are as follows:

- Ca(OH)_2_ alone (35%);
- AgNPs (0.06%) + Ca(OH)_2_;
- AgNPs (0.04%) + Ca(OH)_2_;
- AgNPs (0.03%) + Ca(OH)_2_;
- AgNPs 0.06%;
- AgNPs 0.04%;
- TAP (1 mg/mL);
- Cells alone as a negative control.

To prepare the silver nanoparticle medicaments, 0.5% carboxymethyl cellulose (CMC) was dispersed in distilled water with a magnetic stirrer at 120 rpm until a smooth consistency was obtained. Next, AgNPs (size = 20 nm) (Zhengzhou Dongyao Nano Materials Co., Ltd., Zhengzhou, China) were diffused in the prepared gel with gentle stirring for 1 h. After that, the samples were placed in a sonicator for 2 h. Finally, the medicaments were refrigerated (4 °C) for 4 h to achieve homogeneous gels without entrapped air. The manufacturer already characterized the physical properties of these AgNPs.

The 35% Ca(OH)_2_ paste was prepared by levigating pure Ca(OH)_2_ powder (Somatco, Riyadh, KSA) with a dense aqueous vehicle (propylene glycol: glycerin 1:1) until a paste-like consistency was achieved. Viscous vehicles allowed the slow and extended release of Ca++ and OH^−^ ions [105].

Preparation of TAP (Conc. 1 mg/mL) was performed by dissolving Ciprofloxacin, Minocycline, and Metronidazole (Xi’an Sgonek Biological Technology Co., Ltd., Shaanxi, China), at a ratio of 1:1:1 in 1 mL distilled water. Then, stirring was continued using a magnetic stirrer until completely dissolved.

### 4.3. Cell Culture and Dentin Disk Preparation

As a model for human bone marrow stem cells (hBMSCs), A telomerized human mesenchymal stem cell line (hMSC-TERT) was employed in this study [70]. This cell line was obtained from the stem cell unit, anatomy department, College of Medicine, King Saud University and was generated by overexpression of the human telomerase reverse transcriptase gene (hTERT) [70,71]. Cells were cultured in basal medium (Dulbecco’s Modified Eagle medium, DMEM), which contains 4mM L-glutamine, 4500 mg/L D-glucose, and 110 mg/L 10% sodium pyruvate, and supplemented with 10% fetal bovine serum (FBS), 1% non-essential amino acids and 1% penicillin-streptomycin (All acquired from Thermo-Fisher Scientific Life Sciences, Waltham, MA, USA). Cells were kept in a 5% CO_2_ incubator at 37 °C and 95% humidity, and the medium was changed every 2 days. After reaching 80–90% confluency, cells were treated with 0.025% trypsin (Gibco), sub-cultured in flasks, or used in experiments. An automated cell counter (TC10^TM^, Bio-Rad, Hercules, CA, USA) was used to determine the cell count before seeding. 

For the attachment assay, dentin disks with 1 mm thickness were prepared from extracted human teeth following a previous protocol [20,48]. Briefly, human teeth were freshly extracted and stored in thymol. Teeth with cracks, root resorption, previous root canal treatment, and calcified canals were excluded. A surgical blade was used to remove gingival tissues. Then, a precision saw at 1000 rpm (IsoMet 1000 Precision Saw, IsoMet, Buehler, IL, USA) was used to section the teeth after embedding them in ortho-resin. Disks with 1 mm thickness were obtained, washed with 17% EDTA in an ultrasonic bath, and rinsed with sterile saline. Lastly, they were sterilized using gamma radiation at 25 kilos Gray [106].

For SEM, cells were cultured on treated dentin disks. For the remaining assays, medicaments were applied to cell culture inserts (Falcon^®^, Corning, NY, USA) with a 0.4 μm pore size. This allowed the medicaments to diffuse into the media without direct contact between the cells and any insolubilized particles [107].

### 4.4. Cell Morphology and Attachment using Scanning Electron Microscopy (SEM)

Dentin disks were utilized in this assay to observe the cellular morphology and attachment to dentin. The disks were placed into 24-well culture plates and randomly allocated to one of the groups. Each sample was immersed in 0.2 mL of the assigned material and was incubated for 7 or 14 days at 37 °C in a humidified atmosphere of 5% CO_2_. After each time period, specimens were washed twice with PBS for 5 min and then with 17% EDTA for 1 min [82]. To prepare for cell seeding, the disks were placed in new culture plates containing basal medium (DMEM) and incubated for 24 h [82]. Then, cells were seeded on the treated dentin disks and cultured for 3 days.

Subsequently, samples were prepared for SEM by fixation in 2.5% glutaraldehyde for 48 h, followed by dehydration using a graded series of ethanol (30%, 50%, 70%, 80%, 90%, and 100%). Specimens were dried with critical point drying, coated with gold, and then observed under a SEM (JSM 6360-LV, JEOL Corp., Peabody, MA, USA) at 2000× magnification. 

### 4.5. Cell Proliferation Assay

Cell proliferation was evaluated using the Alamar Blue assay. At days 1, 3, and 5 of treatment, 10% Alamar blue substrate (Thermo Fisher Scientific Life Sciences, Waltham, MA, USA) was added to each well. Cells were incubated for 3 h in the dark, and the fluorescence (Ex 530 nm/Em 590 nm) was measured using a microplate reader (BioTek Synergy II, BioTek Inc., Winooski, VT, USA).

### 4.6. Live–Dead Imaging

Cell apoptosis was evaluated using the acridine orange/ethidium bromide (AO/EB) method, a fluorescence-based staining technique described previously [108]. At 24 and 48 h of treatment, cells were washed twice with PBS and stained with a dual fluorescent dye containing 100 µg/mL AO and 100 µg/mL EB (Sigma^®^, St. Louis, MO, USA) for 1 min. Images were then taken and assessed using a fluorescence microscope (Eclipse Ti, Nikon Imaging Inc., Tokyo, Japan). The differences in uptake of the dual dye indicate viable (green) and non-viable (red) cells. Percentage of dead to live cells was quantified as described previously [108]. Data are representative of three replicas for each experimental condition, where more than 4 images were captured per condition.

### 4.7. Scratch Migration (Wound healing) Assay

Cells (0.1 × 10^6^/well) were seeded in 12-well plates for 24 h to achieve confluency. A sterile ruler was used to reference the center, and an artificial scratch wound was produced in each well using a sterile 200 µL pipette tip. Each well was washed three times with PBS to remove debris. Medicaments were applied, and the wound closure was assessed at 12, 24, and 36 h using a light microscope (Carl Zeiss Canada, North York, ON, Canada). Images were obtained using Zen software (Carl Zeiss Canada, North York, Ontario, ON, Canada). Then, wound healing area percentages were calculated using ImageJ software (U.S. National Institutes of Health, Bethesda, MD, USA) [109] at three periods: 0 to 12 h, 12 to 24 h, and 24 to 36 h, as previously described [110]. Data are representative of 3 replicas for each experimental condition, where 3 images were captured per condition.

### 4.8. Osteogenic Differentiation Assays

The cells were cultured to 80–90% confluency to assess osteogenic differentiation. Then, the basal culture medium was replaced with an osteoinduction medium. The osteo-medium consisted of DMEM containing 50 μg/mL ascorbic acid (Wako Chemicals GmbH, Neuss, Germany), 10 mM β-glycerophosphate, 0.01 µM calcitriol (1α,25-dihydroxy vitamin D3), and 0.01 µM dexamethasone (Sigma-Aldrich, Munich, Germany), as well as 10% FBS and 1% penicillin-streptomycin.

#### 4.8.1. Quantitative Reverse Transcription Real-Time PCR (qRT-PCR)

On days 5 and 10 of osteoblastic differentiation, total RNA was extracted from cell pellets using the innuPREP RNA Mini Kit (Analytic-Jena, Jena, Germany). The concentration and quality of the extracted RNA were measured using the NanoDrop^TM^ 2000 spectrophotometer (ThermoFisher Scientific), and complementary DNA (cDNA) was synthesized using the high-capacity cDNA reverse transcription kit (ThermoFisher Scientific) according to the manufacturer’s instructions. Expression levels of Transforming growth factor beta 1 (TGF-β1), Runt-related transcription factor (Runx2), Alkaline Phosphatase (ALP), and Osteocalcin (OCN) were evaluated using qRT-PCR. Each PCR was carried out in triplicate on a ViiA^TM^ 7 Real-Time PCR System (ThermoFisher Scientific), using a Fast-SYBR Green master mix (ThermoFisher Scientific). Data were normalized to the housekeeping gene (glyceraldehyde-3-phosphate dehydrogenase GAPDH), and the relative gene expression was calculated using the delta-delta CT method. Primer sequences used in this study are listed in Table 1.

#### 4.8.2. Quantification of Alkaline Phosphatase (ALP) Activity

ALP activity was quantified using the ALP Activity Colorimetric Assay Kit (BioVision, Inc., Milpitas, CA, USA) with some modifications. The Cells were cultured in 24-well plates. On days 5 and 10 of osteoblastic differentiation, the wells were rinsed with PBS and fixed with a fixative solution (3.7% formaldehyde in 90% ethanol) for 30 s. The fixative was removed and replaced with a p-nitro-phenyl phosphate solution, and the cells were incubated in the dark for 1 h at room temperature. The optical density for each well was measured at 405 nm using a microplate reader (SpectraMax^®^ M5, Molecular Devices, San Jose, CA, USA). ALP enzyme activity was normalized to the cell count.

#### 4.8.3. ALP Staining

On days 5 and 10 of osteoblastic differentiation, cells were washed with PBS and fixed using an acetone/citrate buffer fixative for 5 min at 25 °C. The stain was prepared by dissolving 2 mg Naphthol-AS-TR-phosphate (Sigma) in 10 mL H2O and 10 mg Fast Red (Sigma) in 12 mL 0.1 Tris buffer. Then, the fixative was replaced with the Naphthol/Fast red stain (1:1), and cells were stained for 1 h at room temperature. Cells were then rinsed 3 times with water and counterstained using Mayer’s hematoxylin solution for 3–4 min at room temperature. Specimens were washed with water, kept in PBS, and observed using a light microscope.

### 4.9. Statistical Analysis

All numerical data are presented as the mean ± SEM. Statistical analysis was performed using one and two-way analysis of variance [ANOVA] with GraphPad Prism 9 software (GraphPad Software, San Diego, CA, USA), and significance was set at (*p* < 0.05). Tukey’s post hoc was used for multiple comparisons.

## 5. Conclusions

In conclusion, compared with Ca(OH)_2_ and TAP, AgNPs have positive effects on cell proliferation, viability, attachment, migration, and osteoblastic differentiation potential. These results highlight the potential application of AgNPs combined with Ca(OH)_2_ or alone as a biomaterial for various clinical applications, such as intracanal medicaments in root canal treatments and endodontic regeneration. However, given the limitations of in vitro studies, further in vivo investigations are needed to deem these medicaments ready for clinical application.

## Figures and Tables

**Table 1:** List of primer sequences used in qRT-PCR.

Primer	Forward	Reverse
TGF-β1	5′-GCAGAGCTGTGAAGCCTTGAGA-3′	5′-TGCCTTCCTGTTGACTGAGTTG-3′
ALP	5′-GACGGACCCTCGCCAGTGCT-3′	5′-AATCGACGTGGGTGGGAGGGG-3′
OCN	5′-GGCAGCGAGGTAGTGAAGAG-3′	5′-CTCACACACCTCCCTCCTG-3′
RUNX2	5′GTA GAT GGA CCT CGG GAA CC3′	5′GAG GCG GTC AGA GAA CAA AC3′
GAPDH	5′-GAAGGTGAAGGTCGGAGT-3′	5′-GAAGATGGTGATGGGATTTC-3′

## Data Availability

The data presented in this study are available on request from the corresponding author.
